# Comparison of Vitamin D Serum Values between Rheumatoid Arthritis and Lupus Populations: An Observational Study

**DOI:** 10.2174/1874312901812010124

**Published:** 2018-08-29

**Authors:** Sahebari Maryam, Elham Atabati, Ravanshad Yalda

**Affiliations:** 1 Rheumatic Diseases Research Center, School of Medicine, Mashhad University of Medical Sciences, Mashhad, Iran; 2 Cellular and Molecular Research Center, School of Medicine, Birjand University of Medical Sciences, Birjand, Iran; 3 Clinical Research Development Unit, Ghaem Hospital, School of Medicine, Mashhad University of Medical Sciences, Mashhad, Iran

**Comparison of Vitamin D Serum Values between Rheumatoid Arthritis and Lupus Populations: An Observational Study**

The Open Rheumatology Journal, **2018**, 12: 65-69

The correct conclusion which is mentioned below:

**Since VitD deficiency is very common in Iran, physiologic doses of VitD supplementation in patients lead to higher serum levels of VitD. Lower VitD values in lupus patients compared with RA ones may stem from intestinal malabsorption, higher doses of corticosteroid therapy, renal involvement and proteinuria, different polymorphisms of VitD receptors, and more sun protection strategies in lupus patients.**

The original conclusion provided was: 

**VitD serum values were lower in lupus patients than RA ones. Since VitD deficiency is very common in Iran, physiologic doses of VitD supplementation in patients lead to higher serum levels of VitD. Lower VitD values in lupus patients compared with RA ones may stem from intestinal malabsorption, higher doses of corticosteroid therapy, renal involvement and proteinuria, different polymorphisms of VitD receptors, and more sun protection strategies in lupus patients.**

